# Neuron-recognizable characteristics of peptides recombined using a neuronal binding domain of botulinum neurotoxin

**DOI:** 10.1038/s41598-022-09145-5

**Published:** 2022-03-23

**Authors:** Hye Rin Kim, Younghun Jung, Jonghyeok Shin, Myungseo Park, Dae-Hyuk Kweon, Choongjin Ban

**Affiliations:** 1 Department of Integrative Biotechnology, Sungkyunkwan University, Seoburo 2066, Suwon, Gyeonggi 16419 Republic of Korea; 2 Institute of Biomolecule Control, Sungkyunkwan University, Seoburo 2066, Suwon, Gyeonggi 16419 Republic of Korea; 3 Biologics Research Center, Sungkyunkwan University, Seoburo 2066, Suwon, Gyeonggi 16419 Republic of Korea; 4 Interdisciplinary Program in BioCosmetics, Sungkyunkwan University, Seoburo 2066, Suwon, Gyeonggi 16419 Republic of Korea; 5 Carl R. Woese Institute for Genomic Biology, University of Illinois at Urbana-Champaign, Urbana, IL 61801 USA; 6 Environmental Health Sciences, School of Public Health, University of Minnesota, Saint Paul, MN 55108 USA; 7 Department of Environmental Horticulture, University of Seoul, 163 Seoulsiripdaero, Dongdaemun-gu, Seoul, 02504 Republic of Korea

**Keywords:** Peptides, Drug delivery, Medical imaging, Biomaterials - proteins, Drug delivery

## Abstract

Recombinant peptides were designed using the *C*-terminal domain (receptor binding domain, RBD) and its subdomain (peptide A2) of a heavy chain of botulinum neurotoxin A-type 1 (BoNT/A1), which can bind to the luminal domain of synaptic vesicle glycoprotein 2C (SV2C-LD). Peptide A2- or RBD-containing recombinant peptides linked to an enhanced green fluorescence protein (EGFP) were prepared by expression in *Escherichia coli*. A pull-down assay using SV2C-LD-covered resins showed that the recombinant peptides for CDC297 BoNT/A1, referred to EGFP-A2ʹ and EGFP-RBDʹ, exhibited ≥ 2.0-times stronger binding affinity to SV2C-LD than those for the wild-type BoNT/A1. Using bio-layer interferometry, an equilibrium dissociation rate constant (*K*_D_) of EGFP-RBDʹ to SV2C-LD was determined to be 5.45 μM, which is 33.87- and 15.67-times smaller than the *K*_D_ values for EGFP and EGFP-A2ʹ, respectively. Based on confocal laser fluorescence micrometric analysis, the adsorption/absorption of EGFP-RBDʹ to/in differentiated PC-12 cells was 2.49- and 1.29-times faster than those of EGFP and EGFP-A2ʹ, respectively. Consequently, the recombinant peptides acquired reasonable neuron-specific binding/internalizing ability through the recruitment of RBDʹ. In conclusion, RBDs of BoNTs are versatile protein domains that can be used to mark neural systems and treat a range of disorders in neural systems.

## Introduction

Botulinum neurotoxins (BoNTs) are lethal toxins produced by bacteria of the genus *Clostridium* (e.g., *Clostridium botulinum*) under anaerobic conditions that can be fatal to humans even at extremely small doses^1^. Among the seven serotypes (A‒G), BoNT A types (BoNT/As) are commonly used commercially as therapeutic and cosmetic agents to treat neuromuscular diseases or autonomic neuronal transmission disorders^2^. BoNT/A includes 10 different subtypes (A1‒A10), which are classified according to the different amino acid sequences. The BoNT/A1 has been characterized in previous research and is an ingredient for medicinal treatments or cosmetics^3^. A *C*-terminus domain of BoNT/A1, a receptor-binding domain (RBD), helps make the toxins bind to the synaptic neurons before endocytosis. The binding onto the neurons is initiated by interactions between the RBD and polysialo-ganglioside complexes on the outer leaflet of a presynaptic lipid bilayer membrane^4^. This preliminary interaction is reinforced by secondary binding to a specific receptor in the presynaptic membrane, synaptic vesicle glycoprotein 2C (SV2C)^5^, resulting in the rapid entry of the toxins into neurons via synaptic vesicles^6^. The peptide A2, which is a residue included in the RBD, having a 14-amino acid sequence, is essential for the toxin to bind to the luminal domain of SV2C (SV2C-LD)^7^. The peptide A2 itself, which is present as a protein residue with 14-amino acids’ sequence, inhibits the endocytosis of the whole sequence BoNT/A1.

The SV2 family is comprised of three paralogues: SV2A, SV2B, and SV2C^8^. Each paralogue has 12 transmembrane domains, with cytosolic *N*- and *C*-termini, and a highly *N*-glycosylated intraluminal loop between transmembrane domains 7 and 8^9^. The three paralogues share 61‒64 and 80% of the sequence and structural homologies, respectively^10^. These SV2 proteins are found in every neurosecretory vesicle in the human body^8^ and are expressed in the peripheral nervous system and neuroendocrine cells^10,11^. Among these paralogues, SV2C expression is most limited, localizing to evolutionarily old regions, with strong expression throughout the striatum, midbrain (particularly in the *substantia nigra*), hindbrain, and ventral pallidum and weak expression in the neocortex, cerebrum, olfactory bulb, hippocampus, and cerebellum^8,12–14^. In particular, SV2C is expressed in 70% of dopaminergic neurons and strongly in certain GABAergic cell types (such as Purkinje cells and medium spiny neuronal cells of cerebellum and striatum, respectively), as well as a fraction of cholinergic neuronal cells^13,15,16^. SV2B and SV2C are expressed in all motor neurons, whereas SV2A is expressed selectively in slow motor neurons^17^. Moreover, all three paralogues are expressed in the pancreas, especially SV2A and SV2C, which are expressed on the insulin-containing granules and participate in glucose-evoked insulin release^18^. Compared to SV2A and SV2B, there have been few detailed studies involved in the expression of SV2C^19^.

Existing pharmacological treatments to neurological disorders, rely on systemic drug administration, result in broad drug distribution with reduced efficacy and an increased risk of side effects^20,21^. The use of systems for targeting specific body parts is a possible option to overcome the critical drawbacks. With this respect, SV2C can be a target protein for treating various diseases originating from disorders of SV2C-incorporating cells and body parts. Indeed, SV2C is a therapeutic target protein for treating Parkinson’s^14,16,22^ and Huntington’s^23^ diseases, temporal lobe epilepsy, hippocampal sclerosis^24^, addiction^25^, and neuropsychiatric conditions^26^. BoNT/A1 has binding affinity to SV2C on motoneurons^17^ and insulin-containing pancreatic granules^18^ and has been used to treat various neurological disorders, including cerebral palsy, hyperhidrosis, migraine, strabismus, and peripheral neuropathy^5–7^. Therefore, anti-SV2C antibodies can be applied to target the body parts for marking or treating the disorders enumerated above. The development and manufacture of antibodies require considerable labor, time, and money because antibodies are often expressed and functionalized in animal bodies or animal cells as the hosts^27–30^. In contrast, relatively short peptides and protein domains are produced easily and inexpensively in bacterial cells, using only simple genomic engineering procedures with no animal ethics issues^31^. At this point, peptide A2 and RBD of the BoNT/A1 prepared using *E. coli* are applicable as a part of efficacious SV2C-targeting or -marking systems.

In this study, recombinant peptide-A2 and RBD connected to an enhanced green fluorescent protein (EGFP) as fluorescent probes were prepared using genetically engineered *E. coli* to confirm their SV2C-binding potential, i.e., the neuro-recognizability. The recombinant peptide-A2/RBD binding affinities to SV2C-LD were estimated by a protein level test using SV2C-LD-binding resins. The association/dissociation kinetics to SV2C-LD were analyzed quantitatively using bio-layer interferometry. The adsorption or internalization to simulated neurons, differentiated PC-12 cells, was validated by confocal laser fluorescence microscopy (CLFM) and flow cytometry.

## Results

### Production of the neuro-recognizable recombinant peptides

**Figure 1:**
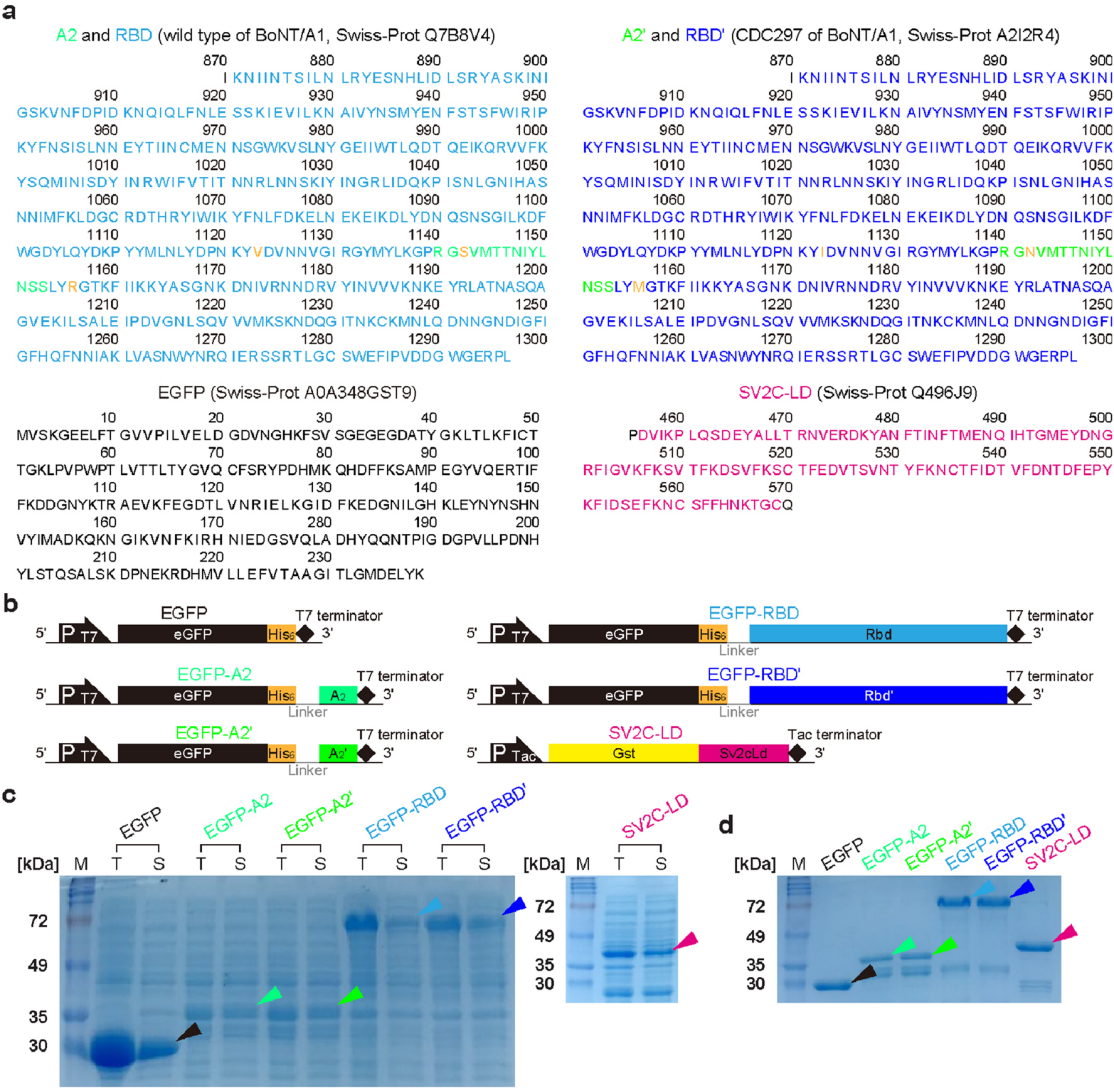
Preparation of the recombinant peptides. (**a**) Gene sequences for the based peptides used for constructing the recombinant peptides (EGFP, EGFP-A2, EGFP-A2ʹ, EGFP-RBD, EGFP-RBDʹ, and SV2C-LD). A2 peptide and receptor binding domain (RBD) of wild-typed botulinum neurotoxin A subtype 1 (BoNT/A1; Swiss-Prot Q7B8V4: 1140‒1153 and 871‒1296); A2ʹ peptide and RBDʹ of CDC5228 BoNT/A1 (Swiss-Prot A2I2R4: 1140‒1153 and 871‒1296), enhanced green fluorescent protein (EGFP; Swiss-Prot A0A348GST9, 1‒239), and luminal domain of synaptic vesicle glycoprotein 2C (SV2C-LD; Swiss-Prot Q496J9: 456‒569). Linker sequence: GGGGS. (**b**) Schematic structural illustrations of the gene constructs (eGFP-His_6_, eGFP-His_6_-linker-A_2_, eGFP-His_6_-linker-A_2_ʹ, eGFP-His_6_-linker-Rbd, eGFP-His_6_-linker-Rbdʹ, and Gst-Sv2cLd) used for expressing the recombinant peptides (EGFP, EGFP-A2, EGFP-A2ʹ, EGFP-RBD, EGFP-RBDʹ, and SV2C-LD). Gel images of the recombinant peptides in the SDS-PAGE, obtained after (**c**) the expression and (**d**) purification procedures (*M* marker peptide, *T* Total peptide, *S* soluble peptide; arrows indicate the target peptides). Original gels are presented in Supplementary Fig. S1.

Two different peptide A2s and RBDs of BoNT/A1, wild-type (ATCC3502; A2 and RBD) and CDC297 [having a same DNA sequence with CDC1903, CDC5328, and CDC51303 (*ha* − /*orfX* + A1 strains)]; A2ʹ and RBDʹ], were compared, and the better in terms of the neuro-recognizability was chosen. In particular, only three amino acid sequences at 1123, 1142, and 1156 of two RBDs are different [RBD: valine (V), serine (S), and arginine (R); RBDʹ: isoleucine (I), asparagine (N), and methionine (M)] (Fig. 1a). Only the third amino acids of A2 and A2ʹ were different as serine and asparagine, respectively. EGFP was connected to the *N*-termini of the peptides through a His6-tag (6 histidine) and a linker to mark these SV2C-LD-bindable peptides (Fig. 1b). The His6-tag and linker were used for purification and flexibility/folding, respectively, and a glutathione *S*-transferase (GST)-tag of SV2C-LD was adopted for the pull-down assay. Based on the designed constructs, expected molecular weights of His6-tagged EGFP, EGFP-A2, EGFP-A2ʹ, EGFP-RBD, EGFP-RBDʹ, and GST-tagged SV2C-LD are 27.77, 29.61, 29.64, 77.77, 77.78, and 38.88 kDa, respectively. Accordingly, the thick and dark bands with the corresponding sizes were observed clearly in all the sodium dodecyl sulfate (SDS)-PAGE gel lanes of the total and soluble recombinant peptides expressed (Fig. 1c). After purification using Ni^2+^-nitrilotriacetic acid- (Ni–NTA) and glutathione agarose resins, thick and dark target bands were observed clearly, while many non-target bands were disappeared or became vague (Fig. 1d). Unfortunately, some non-target bands remained in the gel lanes of EGFP-A2 and EGFP-A2ʹ, which might contribute to the proteolyzed residues. Consequently, the constructs of EGFP-A2 and EGFP-A2ʹ were optimized in the section below. On the other hand, the remaining peptides (EGFP, EGFP-RBD, EGFP-RBDʹ, and SV2C-LD) were used in further experiments with the present forms.

### SV2C-LD-binding affinity of the neuro-recognizable recombinant peptides

**Figure 2:**
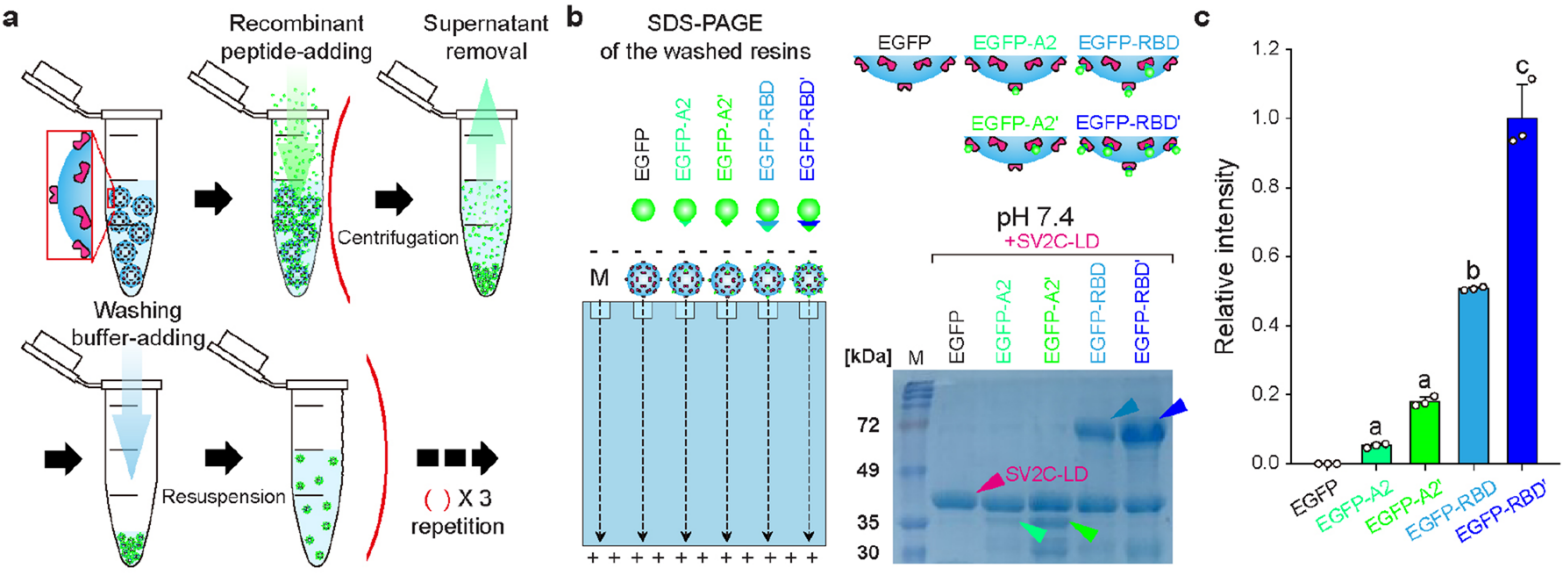
Characterization of SV2C-LD-binding affinity of the recombinant peptides using the GST-pull-down assay. (**a**) Schematic representation of the pull-down assay and the expected status of the resins after the assay. (**b**) Schematic illustration and gel image of the neuro-recognizable recombinant peptides (EGFP, EGFP-A2, EGFP-A2ʹ, EGFP-RBD, and EGFP-RBDʹ) in the SDS-PAGE, obtained after the pull-down assay (M, marker peptide; arrows, except a pink arrow, indicate the target neuro-recognizable peptides). (**c**) Relative amounts of the target neuro-recognizable peptides measured using the ImageJ processing with the gel images, based on the concept that the amount of EGFP-RBDʹ is 1. Data with different letters a‒c in the plot represent significant differences according to the Tukey’s test (*n* = 3; average ± s.d.; *P* < 0.05). Original gels are presented in Supplementary Fig. S1.

The SV2C-LDs were immobilized onto glutathione agarose resins via a GST-tag at the *N*-terminus to estimate the binding affinity of the fabricated recombinant peptides to SV2C-LD (Fig. 2a). The recombinant peptides were first incubated with the SV2C-LD-immobilized resins to allow time for binding, and the unbound recombinant peptides were washed out. After the washing step, the resins binding the recombinant peptides were loaded and electrophoresed in an SDS-PAGE gel (Fig. 2b). As shown in the gel image, the SV2C-LD bands were found not only in all the gel lanes, but the bands for the target recombinant peptides were also detected in the respective lanes of EGFP-A2, EGFP-A2ʹ, EGFP-RBD, and EGFP-RBDʹ, whereas none of the bands for EGFP was observed. This result shows none of the binding affinities of EGFP. In addition, the bands of EGFP-A2ʹ and EGFP-RBDʹ were thicker and darker than those of EGFP-A2 and EGFP-RBD, respectively, indicating the greater binding affinities of CDC297-originated peptides (EGFP-A2ʹ and EGFP-RBDʹ) than those of the wild-type peptides (EGFP-A2 and EGFP-RBD). This result is consistent with the relative intensity of the target bands (Fig. 2c). The values of EGFP-A2ʹ and EGFP-RBDʹ were ~ 3.43- and ~ 1.97-times larger than those of EGFP-A2 and EGFP-RBD, respectively, showing the greater binding affinities of the CDC297-originate peptides. In addition, the values of EGFP-RBD and EGFP-RBDʹ were ~ 9.66- and ~ 5.54-times higher than those of EGFP-A2 and EGFP-A2ʹ, respectively. This result suggests that the entire sequences of the RBDs bind better to SV2C-LD than the fragments (peptide A2s) of RBDs. In other words, the RBD sequences subtracted from the peptide A2s have a positive effect on the binding affinity. Consequently, EGFP-A2ʹ and EGFP-RBDʹ, the CDC297-originated peptides, were used in further experiments because of the greater binding affinities to SV2C-LD.

### SV2C-LD-association and -dissociation of the neuro-recognizable recombinant peptides

**Figure 3:**
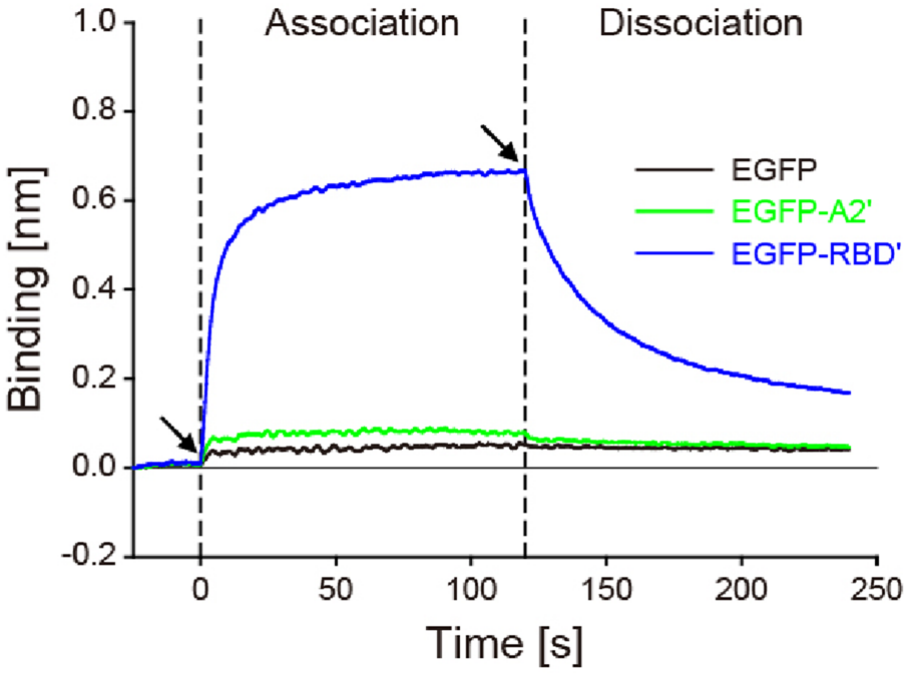
Characterization of SV2C-LD-association/dissociation of the recombinant peptides using bio-layer interferometry. Left and right arrows indicate the initial points of association and dissociation, respectively.

The association and dissociation of the recombinant peptides to SV2C-LD were quantified by first immobilizing the GST-tagged SV2C-LDs onto well plates coated with anti-GST antibodies. While monitoring the bio-layer interferometric signals, the recombinant peptides were added to the SV2C-LD-immobilized wells to measure the association kinetics of the peptides and washed away with fresh buffer solutions to determine the dissociation kinetics (Fig. 3). The binding thicknesses of EGFP, EGFP-A2ʹ, and EGFP-RBDʹ were increased to 0.050, 0.071, and 0.657 nm within 120 s for the association, respectively. The values of EGFP, EGFP-A2ʹ, and EGFP-RBDʹ were then decreased to 0.015, 0.032, and 0.499 nm within the subsequent 120 s for the dissociation, respectively. The kinetic constants, *k*_a_, *k*_d_, and *K*_D_, were calculated based on the curves for the change in value (Table 1). The *k*_a_ values of EGFP-A2ʹ and EGFP-RBDʹ were 29.46- and 201.76-times larger than that of EGFP as 5.26 × 10^3^ and 7.69 × 10^2^ M^−1^ s^−1^, respectively, which suggests their association properties specific to SV2C-LD. The *k*_d_ value of EGFP-A2ʹ (6.56 × 10^−2^ s^−1^) was 5.96- and 2.29-times larger than EGFP and EGFP-RBDʹ, respectively, showing the fastest dissociation. The *K*_D_ value of EGFP-RBDʹ was the smallest as 5.45 × 10^0^ μM, which was 33.87- and 15.67-times smaller than those of EGFP and EGFP-A2ʹ. This result shows that EGFP-RBDʹ has strong binding affinity to SV2C-LD, at least in protein-level. In contrast, EGFP-A2ʹ exhibited weak binding affinity, which is in accordance with the GST pull-down assay result.

**Table 1:** Equilibrium dissociation constant (*K*_D_ = *k*_d_/*k*_a_) and apparent association (*k*_a_) and dissociation (*k*_d_) rate constants for the interaction of SV2C-LD with EGFP, EGFP-A2ʹ, and EGFP-RBDʹ, determined from the bio-layer interferometric result.

Peptides	*k*_a_ [M^−1^ s^−1^]	*k*_d_ [s^−1^]	*K*_D_ [μM]	*R*^2^
EGFP	2.61 × 10^1^	4.82 × 10^−3^	1.85 × 10^2^	0.55
EGFP-A2ʹ	7.59 × 10^2^	6.56 × 10^−2^	8.54 × 10^1^	0.94
EGFP-RBDʹ	5.26 × 10^3^	2.87 × 10^−2^	5.45 × 10^0^	0.99

### Endocytosis of the neuro-recognizable recombinant peptides

**Figure 4:**
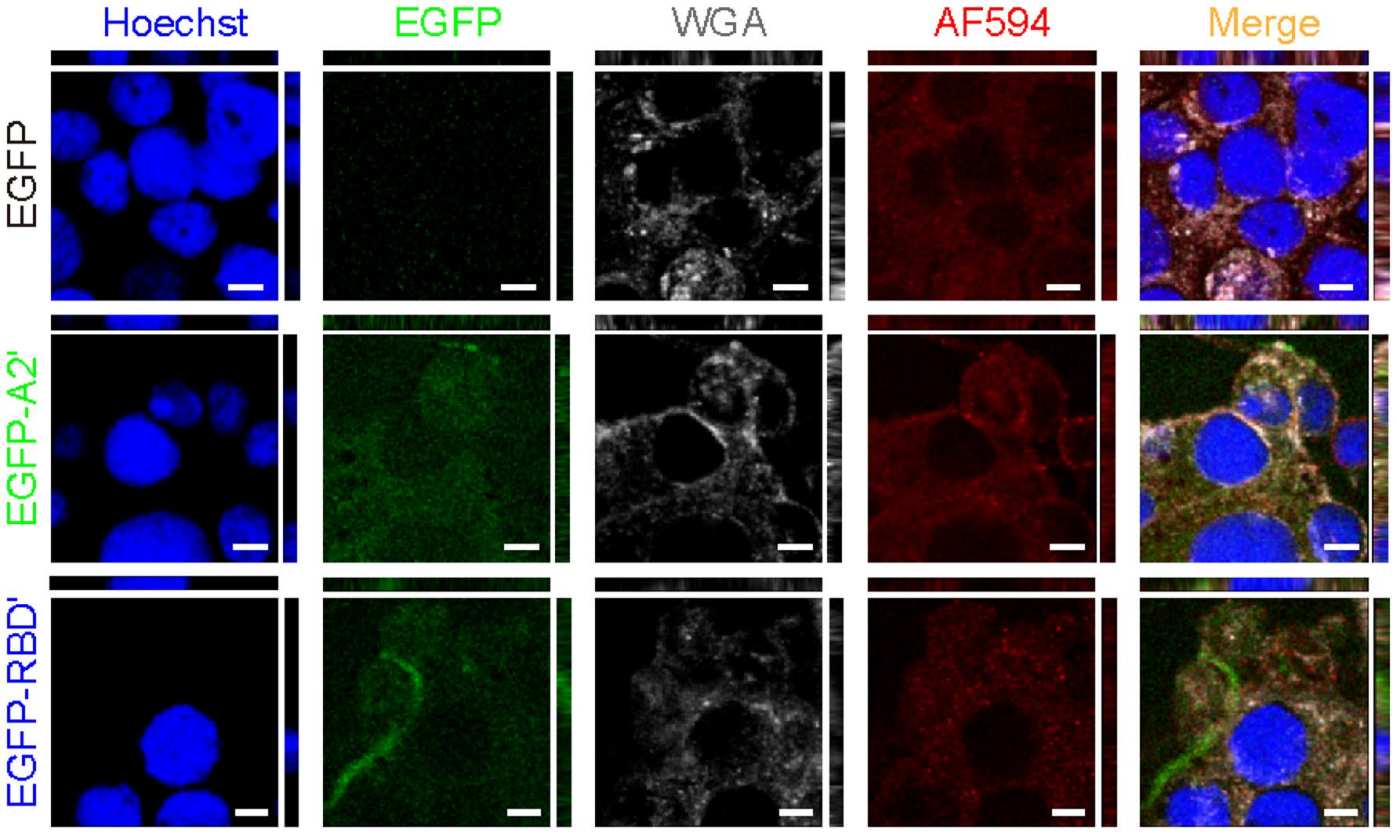
Orthogonal-viewed images obtained using confocal laser fluorescence microscopy. Differentiated PC-12 cells incubated with culture media containing the recombinant peptides (EGFP, EGFP-A2ʹ, and EGFP-RBDʹ) for 1 min (scale bars, 5 μm). Nuclei, EGFP, glycolipids/glycoproteins, and late endosomes in the cells are in blue (Hoechst, Hoechst 33258), green (EGFP), gray (WGA, wheat germ agglutinin Alexa Fluor 647 conjugate), and red (AF594, Alexa Fluor 594), respectively. Images at the bottom-left, top-left, and bottom-right in each image are the upper, y-axis, and x-axis views, respectively.

**Figure 5:**
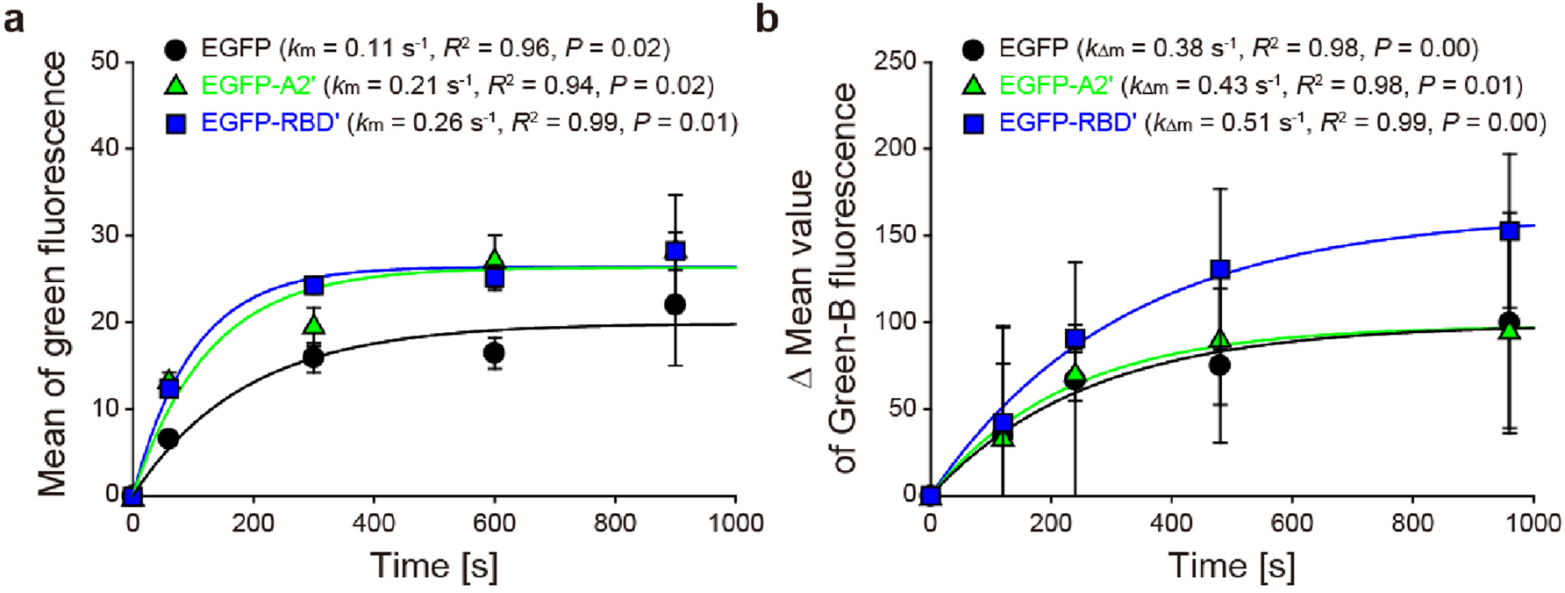
Characterization of adsorption/absorption of the recombinant peptides to differentiated PC-12 cells. (**a**) Mean of green fluorescence in the cells incubated with culture media containing the recombinant peptides (EGFP, EGFP-A2ʹ, and EGFP-RBDʹ), determined using the ImageJ processing of confocal laser fluorescence microscopic images. (**b**) Changes in mean values for Green-B fluorescence signal of the cells incubated with the recombinant peptide-containing media, measured using the flow cytometry. All data represent average ± s.d. (*n* = 3), the fitting curves were regressed with the equation [$f\left(t\right)=a\left(1-{b}^{t}\right)$; $a>0$ and $0<b<1$], and the adsorption/absorption rate constants (a, ${k}_{\mathrm{m}}$; b, ${k}_{\Delta \mathrm{m}}$) were determined from the equation as ${k}_{\mathrm{m}}\mathrm{ and }{k}_{\Delta \mathrm{m}}={f}^{^{\prime}}\left(0\right)=-a\mathrm{ln}b$.

The cell-level neuro-selective recognition abilities of EGFP, EGFP-A2ʹ, and EGFP-RBDʹ were verified using neuron-like differentiated PC-12 cells, known for expressing SV2s^32,33^. For CLFM (Fig. 4, Supplementary Figs. S2a, S3), unlike EGFP, which did not contain a neuro-recognizable residue, the green-fluorescent signals of EGFP-A2ʹ and EGFP-RBDʹ were observed in/on PC-12 cells only 1 min after incubation. This result indicates the faster endocytosis of EGFP-A2ʹ and EGFP-RBDʹ than EGFP, resulting from neuro-specifically recognizable residues, A2ʹ and RBDʹ. After 5 min incubation, the green signals were observed in all PC-12 cells treated with either EGFP, EGFP-A2ʹ, or EGFP-RBDʹ (Figs. S2b, S3). The green signals of EGFP-A2ʹ were negligible in the Mardin–Darby canine kidney (MDCK) cells incubated for 1 min (Figs. S4a, S5). Five minutes after incubation, weak green cell signals were observed in the images for all recombinant peptides (Fig. S4b). However, for all recombinant peptides, the green cell signals of MDCK cells at 5 min were significantly smaller than those of differentiated PC-12 cells at 5 min (Fig. S5). This indicates the diffusional internalization non-specific to MDCK cells and indirectly implies the internalization of EGFP-A2ʹ and EGFP-RBDʹ specific to PC-12 cells. Unfortunately, differences in the green endocytic signals among the recombinant peptides are difficult to discriminate by the naked eye. Therefore, the changes in the endocytic green-fluorescent signal were quantified based on the CLFM images over time using the image processing technique, and the kinetic rate constants (${k}_{\mathrm{m}}$) were analyzed using the regression fitting curves (Fig. 5a). The ${k}_{\mathrm{m}}$ value of EGFP-RBDʹ was the largest at 0.26 s^−1^, which showed 2.49- and 1.26-times faster endocytosis than those of EGFP and EGFP-A2ʹ (0.11 and 0.21 s^−1^), respectively.

In addition to CLFM, flow cytometry was conducted to validate the endocytosis of the green-fluorescent peptides (Fig. S6a,b). The mean values of the Green-B channel fluorescence in the cells were acquired based on the histograms (Fig. S6c). The mean values subtracting the value of the control group were recorded as the ‘$\Delta mean$ values’ and analyzed using the regression fitting curves to determine the kinetic rate constants (${k}_{\Delta \mathrm{m}}$) (Fig. 5b). Like the trend in CLFM, a ${k}_{\Delta \mathrm{m}}$ value of EGFP-RBDʹ was the highest at 0.51 s^−1^, indicating a 1.36- and 1.18-times faster endocytosis than those of EGFP and EGFP-A2ʹ at 0.38 and 0.43 s^−1^, respectively. Despite the discrepancies in the individual value in the two measurements (CLFM and flow cytometry), the tendencies for endocytosis were the same regardless of the measurement. These are also in agreement with the biolayer interferometry above. Overall, both EGFP-A2ʹ and EGFP-RBDʹ can bind selectively to the SV2C-LDs present in the outer membrane of neurons and internalize them into the neurons. Moreover, the neuro-recognizable efficacy of EGFP-RBDʹ is greater than that of EGFP-A2ʹ.

## Discussion

The crystal structure of RBD in complex with SV2C-LD reveals dominating backbone-backbone interactions between two short β-sheet, involving residues around E556‒F563 of SV2C and R1140‒N1147 of RBD. Additionally, the co-crystal structure suggests that a cation-π stacking interaction between F563 of SV2C and R1156 of RBD is crucial for binding^4^. In this concept, mutations in R1140‒N1147 or R1156 of RBD can critically influence the binding affinity of RBD to SV2C-LD. Indeed, mutating R1156 of RBD (R1156E) significantly decreased the binding^7^. In present study, only three amino acid sequences at 1123, 1142, and 1156 are different between RBD (V, S, and R) and RBDʹ (I, N, and M). The difference between RBD and RBDʹ at 1123 (V1123I) is known neither as a key amino acid of ganglioside binding site nor protein receptor binding site^5^. Therefore, among three sequence differences between RBD and RBDʹ (V1123I, S1142N, and R1156M), S1142N and R1156M can greatly affect the binding affinity. With this respect, the greater binding affinity of RBDʹ than RBD was verified in this study.

Through binding of the A2ʹ and RBDʹ residues, EGFP-A2ʹ and EGFP-RBDʹ exhibited specific binding affinity to SV2C-LD decorating the outer membrane of differentiated PC-12 cells, resulting in endocytosis. In particular, in EGFP-RBDʹ, the stronger SV2C-LD-binding affinity and greater endocytosis were verified compared to those of EGFP-A2ʹ. Therefore, EGFP-RBDʹ by itself could be a possible marker to various SV2C-occurring cells, such as dopaminergic, Purkinje, medium spiny, cholinergic, and motor neurons^13,15–17^. In addition, if the EGFP residue of EGFP-RBDʹ is displaced with other residues having bioactivities in neurons, this recombinant construct could be applied to treat a range of neuronal malfunctions. For example, the removal of K63-linked ubiquitin chains on misfolded α-synuclein accumulates by deubiquitinase Usp8 may cause α-synuclein accumulation in Lewy body disease^34^. The RBDʹ constructs connected to the ubiquitination-upregulating or deubiquitination-downregulating enzymes might be a possible medication for the Parkinson’s disease. In this manner, the RBDʹ might be a versatile neuro-recognizable residue for marking and targeting the SV2C-LD-containing cells. Moreover, the RBDʹ constructs can be produced easily and abundantly in *E. coli*, without animal ethics issues, using simple protein engineering techniques^31^, which can lower the production costs.

Synaptic vesicle-dependent endocytosis is well known for its fast procedures^35–37^. For the kiss-and-run, clathrin-mediated endocytosis, and bulk ultrafast endocytosis and endosomal budding models, the spending times for endocytosis are 1‒2, 15‒20, and ~ 0.1 s, respectively^38^. Therefore, the synaptic vesicle-targeting endocytosis has a great potential for neuron-specific delivery of various neuroactive molecules. This is also in accordance with the PC-12 endocytosis data of the RBDʹ recombinant peptides via fast and strong binding to SV2C-LD in the present study. In previous study, the *K*_D_ value of wild-type RBD to SV2C-LD was 0.26 μM^7,39^. In present study, the greater binding affinity of RBDʹ over that of wild-type RBD was verified. Therefore, the *K*_D_ value of RBDʹ was < 0.26 μM, which is a reasonable binding affinity.

The *K*_D_ value of RBDʹ was higher than *K*_D_ values (1.1‒5400 pM) of various monoclonal antibodies anti-targeting proteins^40^. Moreover, the *K*_D_ value was increase ~ 21-times to 5.45 μM because of the addition of EGFP-, polyhistidine- (His6-), and linker to the *N*-terminus of RBDʹ (EGFP-RBDʹ). Such binding affinity can be improved by editing the structure and some key amino acids of RBDʹ or recruiting other RBDs from other subtypes of BoNT/A. Fortunately, there are still synaptic vesicle proteins that can be targeted by the other RBDs, such as SV2A, SV2B, and synaptotagmins instead of SV2C^41^. In particular, synaptotagmins are ~ 9-times more incorporated in one average synaptic vesicle than the SV2 family in terms of the number of their copies^42^. After the recombinant peptides are optimized in terms of neuron-recognizibility, the endosomal escape of the peptides could be a further subject for the development of neuro-marking and -targeting systems. This study shows that the RBDs of BoNTs can be used as the block materials of recombinant peptides for marking nerve systems or treating various neuronal diseases.

## Conclusions

Recombinant peptides specifically binding to SV2C-LD were fabricated by recombining EGFP to either peptide A2 (subdomain of RBD) or RBD of BoNT/A1s. The peptide A2 and RBD (A2ʹ and RBDʹ) of CDC297 (Swiss-Plot A2I2R4) had greater binding affinities to SV2C-LD than the wild-type peptide A2 and RBD (Swiss-Plot Q7B8V4), respectively. Among the all recombinant peptides prepared, the *K*_D_ value of EGFP-RBDʹ was the smallest, suggesting the highest binding affinity to SV2C-LD. EGFP-RBDʹ exhibited the fastest and best endocytosis into simulated neurons owing to the relatively reasonable binding affinity. Overall, the recruitment of RBDʹ improved the neuron-specific binding and endocytosis to the recombinant peptides. In conclusion, RBDs of BoNTs are versatile block materials with the potential for marking neural systems and treating various disorders in the neural systems.

## Methods

### Materials

Luria–Bertani broth (LB) medium was obtained from BD Difco (Sparks, MD, USA). Ni–NTA and glutathione-agarose resins were purchased from Thermo Fisher Scientific Inc. (Waltham, MA, USA) and Millipore Co. (Bedford, MA, USA), respectively. Broad-range pre-stained protein biomarker (EBE-1031) was obtained from ELPIS-Biotech. Inc. (Daejeon, Republic of Korea). Isopropyl-β-d-1-thiogalactopyranoside (IPTG), 4-(2-aminoethyl) benzenesulfonyl fluoride hydrochloride (AEBSF), SDS, polyacrylamide, and Tween 20 were procured from Sigma Aldrich Co. (St. Louis, MO, USA). Radio-immuno-precipitation assay (RIPA) buffer (#9806; Cell Signaling Technology, Beverly, MA, USA), bovine serum albumin (BSA), Roswell Park Memorial Institute (RPMI) 1640 media, horse serum, fetal bovine serum (FBS), 10× antibiotic–antimycotic solution, collagen I (from rat tail), and mouse Nerve Growth Factor-2.5S (NGF; murine submaxillary gland) were obtained from GIBCO/Invitrogen (Grand Island, NY, USA) were used as received. Minimum essential medium (MEM), penicillin G, streptomycin, amphotericin B, and phosphate-buffered saline (PBS) were obtained from Hyclone (Logan, UT, USA). All chemicals were of analytical reagent grade.

### Strain and plasmids

Table 2 lists the *E. coli* strain and plasmids used in the present study. Deoxyribose nucleic acid (DNA) sequences having > and ≤ 60 base pairs (bps) were cloned using a ligation-independent cloning method with T4 DNA polymerase (New England Biolabs Inc., Beverly, MA, USA) and a site-directed mutagenesis technique, respectively. Gene amplification using a polymerase chain reaction (PCR) with a Q5 site-directed mutagenesis kit (New England Biolabs Inc.) was repeated 30-times with the following cycles: denaturation (98 ℃, 3 s), elongation (72 ℃, 30 s kbp^−1^), and annealing (55‒61 ℃, 3 s); the first denaturation and final annealing steps were performed for three and three minutes, respectively. Impurities in the final PCR solution were then removed by incubation with Dpn1 (37 ℃ and 1 h) to prevent self-ligation. DNA fragments amplified by PCR were ligated by incubation with T4 DNA polymerase (25 ℃, 2.5 min → 10 min icing). The codons for expressing the BoNT/A1 RBD (Swiss-Prot Q7B8V4 and A2I2R4: 871‒1296), EGFP (Swiss-Prot A0A348GST9: 1‒239), and SV2C-LD (Swiss-Prot Q496J9: 456‒569) were optimized for expression in *E. coli* BL21 (DE3) using the Oligo TM Calculator supplied by Cosmo Genetech (http://www.cosmogenetech.com/cosmo/productmngr/prm01_tm_server.jsp; Seoul, Republic of Korea). Detailed preparation procedures of the primers and plasmid constructs (pET28b-eGFP-His_6_-linker-A_2_, pET28b-eGFP-His_6_-linker-A_2_ʹ, pET28b-eGFP-His_6_-linker-Rbd, pET28b-eGFP-His_6_-linker-Rbdʹ, pET28b-His_6_-A_2_ʹ-linker-eGFP, pET28b-His_6_-eGFP-linker-A_2_ʹ, and pGEX-4 T-1-Sv2cLd) used in this study are described in the Supplementary Information (Table S1, Fig. S7).

**Table 2:** *E. coli* strain and plasmids utilized in this study.

Sort	Name	Description	Reference
*E. coli*	BL21 (DE3)	F^−^ ompT Ion *hsdS*_*B*_(r^−^_B_, m^−^_B_) *gal dcm*	New England Biolabs Inc.
Plasmids	pET28b-eGFP-His_6_	EGFP + 6 × His-tag, T7 promoter, Kan^R^ from *H. sodomense*	Addgene
	pET28b-eGFP-His_6_-linker-A_2_	pET28b-eGFP-His_6_ + linker (GGGGS) + wild-typed (WT) peptide A_2_ from *C*-terminal heavy chain (HC_C_) of *C. botulinum*	This study
	pET28b-eGFP-His_6_-linker-A_2_ʹ	pET28b-eGFP-His_6_ + linker + CDC297 peptide A_2_ from HC_C_ of *C. botulinum*	
	pET28b-eGFP-linker-A_2_ʹ intermediate	Intermediate plasmid for constructing pET28b-His_6_-eGFP-linker-A_2_ʹ	This study
	pET28b-His_6_-eGFP-linker-A_2_ʹ	Constructed plasmid based on pET28b-eGFP-His_6_-linker-A_2_ʹ	This study
	pET28b-A_2_ʹ-linker-eGFP-His_6_ intermediate	1st intermediate plasmid for constructing pET28b-His_6_-A_2_ʹ-linker-eGFP	This study
	pET28b-A_2_ʹ-linker-eGFP intermediate	2nd intermediate plasmid for constructing pET28b-His_6_-A_2_ʹ-linker-eGFP	This study
	pET28b-His_6_-A_2_ʹ-linker-eGFP	Constructed plasmid based on pET28b-eGFP-His_6_	This study
	pET28b-eGFP-His_6_-linker-Rbd	pET28b-eGFP-His_6_ + linker + WT Rbd from HC_C_ of *C. botulinum*	This study
	pET28b-eGFP-His_6_-linker-Rbd^1^ʹ intermediate	1st intermediate plasmid for constructing pET28b-eGFP-His_6_-linker-Rbdʹ	This study
	pET28b-eGFP-His_6_-linker-Rbd^2^ʹ intermediate	2nd intermediate plasmid for constructing pET28b-eGFP-His_6_-linker-Rbdʹ	This study
	pET28b-eGFP-His_6_-linker-Rbdʹ	pET28b-eGFP-His_6_ + linker + CDC297 Rbd from HC_C_ of *C. botulinum*	This study
	pGEX-4T-1	GST-tag, Tac promoter, Amp^R^ from *E. coli*	Addgene
	pGEX-4T-1-Sv2cLd	pGEX-4T-1 + WT Sv2cLd from human	This study

### Expression and purification of the peptides

*E. coli* BL21 cells (DE3) were transformed with the plasmid constructs cloned for the expression of the recombinant peptides and SV2C-LD. A transformed colony was incubated in 10 mL LB media containing antibiotics for 12 h at 4 ℃ with shaking. Six hundred milliliters of LB media containing antibiotics were inoculated 1:100 with the overnight culture. The *E.coli* was treated with IPTG (final concentration, 0.1 mM), expressed for 20 h at 16 ℃ (optical density at *λ* = 600 nm, 0.5‒1.0), and harvested by centrifugation [6000 rotations per minute (rpm), 5 min]. The cell pellets were re-suspended in 10 mL PBS containing 1 mM AEBSF, lysed by sonication in ice, and centrifuged again (13,000 rpm, 30 min). The His6- and GST-tagged peptides present in the supernatants were loaded in columns filled with Ni–NTA and glutathione resins, respectively, which had been pre-equilibrated with an equilibrium buffer comprising 0.05 Tris–HCl and 0.3 M NaCl (pH 8.0). After agitation (30 min at 25 ℃), the resins were washed five times with a one-column volume of washing buffers [pH 8.0; equilibrium buffer + 10% v/v glycerol + either 10 mM imidazole (for Ni–NTA resin) or 10 mM glutathione (for GST-resin)]. The peptides bound on resins were eluted by adding 500 μL of elution buffers [pH 8.0; equilibrium buffer + either 250 mM imidazole (for Ni–NTA resin) or + 250 mM glutathione (for GST-resin)]. The peptides eluted were analyzed by electrophoresis in a 12% w/v polyacrylamide gel containing SDS (SDS-PAGE).

The recombinant peptide structures (Fig. S8) were optimized by SDS-PAGE of the purified peptides using the total harvested peptides and the soluble and insoluble (precipitated) peptides (50 μg) after the centrifugation. Herein, the quantity of peptides was quantified based on a standard of BSA using a Lowry assay (Protein DC kit; Bio-Rad, CA, USA). The intensities of the target bands were quantified under Image J (available as freeware from http://rsb.info.nih.gov/ij/) processing of the gel images and converted to the relative amounts divided by the intensity value of the total harvested His6-A2ʹ-EGFP.

### Solubilizing and refolding of the peptides

Harvested *E. coli* pellets expressing the recombinant peptides were washed three times with cold PBS and lysed in RIPA buffer. The cell lysates were collected by centrifugation [13,000 relative centrifugal force (RCF), 15 min, 4 °C]. To dissolve the precipitates after centrifugation and regain their function, the insoluble peptides were unfolded by dispersing them in 9 mL of an 8 M urea solution in PBS with stirring (4 ℃, 1 h). The unfolded peptides were then centrifuged (13,000 rpm, 30 min), and the supernatants were loaded in a column filled with Ni–NTA resins. The recombinant peptides bound on the resins were washed eight times with the urea solutions to remove urea and allow refolding with no aggregation. The concentration of the urea solutions was lowered gradually from 8 to 0 M. The refolded peptides on the resins were eluted by adding 500 μL of PBS containing 250 mM imidazole (pH 7.4).

### GST pull-down assays

The recombinant peptides to SV2C-LD were accessed using GST pull-down assays. First, purified 15 μM SV2C-LD was immobilized on 50 μL slurry of glutathione agarose resin and agitated with 20 μM of the recombinant peptides in a washing buffer (50 mM sodium phosphate buffer; total volume: 100 μL; pH 7.4, 10 min, 4 ℃). As a washing procedure, the resins binding the peptides were collected by centrifugation (13,200 rpm, 1 min), separated from the supernatants, and re-suspended in 1 mL of fresh washing buffer. The resins washed with three repetitions of this procedure were re-suspended in 50 μL of the washing buffer and mixed with 250 μL of the washing buffer containing 12% w/v SDS. The mixture solutions containing the peptide-binding resins were analyzed by SDS-PAGE. The intensities of the bands for the target recombinant peptides were quantified by Image J processing with the acquired gel image. The intensities of the target bands were normalized based on the concept that the amount of SV2C-LD in a single gel lane is the same as those in the other lanes. The normalized intensities were converted to the relative amounts by dividing by the normalized value of EGFP-RBDʹ.

### Bio-layer interferometry

The protein-level binding interactions between the recombinant peptides and SV2C-LD were measured by biolayer interferometry (BLItz system; FortéBio, Fremont, CA, USA). First, by monitoring the signal, 40 μL of GST-tagged SV2C-LD (40 μM) dispersed in the biolayer interferometry buffer (pH 7.4; 0.05% w/v Tween 20 and 1 mg mL^−1^ BSA in PBS) was dropped and immobilized onto anti-GST biosensors (FortéBio), which was then washed and equilibrated with fresh buffer. Subsequently, by monitoring the signal, 40 μL of the recombinant peptides (20 μM) dispersed in the buffer was dropped onto the SV2C-LD-covered biosensors, followed by washing with fresh buffer. The binding constants, including association rate (*k*_a_), dissociation rate (*k*_d_), and *k*_d_/*k*_a_ (*K*_D_) constants, were calculated from the association and dissociation curves, using the BLItz system software (FortéBio) according to the manufacturer’s instruction.

### Cell culture and differentiation

Before the culture, the bottom surface of the microplate-wells was coated with I-type collagen by adding 1 mL of a collagen solution (50 μg mL^−1^ collagen in an aqueous 20 mM acetic acid solution; 25 °C, 1 h) to attach the cells to the surface. After decanting the collagen solution, the wells were washed three times with RPMI 1640 media. The PC-12 cell line (Korean Cell Line Bank, Seoul, Korea), simulated neurons, was cultured in the collagen-coated wells containing RPMI 1640 media supplemented with 10% v/v heat-inactivated horse serum, 5% v/v heat-inactivated FBS, and 1% v/v 10× antibiotic–antimycotic solution under humidified air conditions (37 °C, 5% CO_2_). The MDCK (purchased from KCLB) cell line was grown in MEM supplemented with 10% v/v heat-inactivated FBS, 100 unit mL^−1^ penicillin G, 100 μg mL^−1^ streptomycin, and 0.25 μg mL^−1^ amphotericin B under humidified air (37 °C, 5% CO_2_). For cell growth, the culture media were replaced every other day with fresh culture media. To differentiate the PC-12 cells, the remaining media were replaced every other day with 100 ng mL^−1^ NGF-added fresh culture media for an additional 5‒7 days. Before the experiments, the cell viability in 1–5 passages was assessed manually using a Trypan Blue dye exclusion test.

### Confocal laser fluorescence microscopy (CLFM)

Coverslips laid on the bottom surface of 12-well microplate were coated twice by serial treatments of the 50 μg mL^−1^ collagen solution, followed by treatment with 1 mg mL^−1^ poly-D-lysine-added RPMI 1640 media (1 h for each treatment at 25 °C). After washing three times with fresh RPMI 1640 media, the collagen/poly-d-lysine-coated coverslips on the plate bottom were stored at 4 °C before use. Harvested PC-12 cells (2 × 10^5^ cells well^−1^) were seeded and adhered to the coverslips and differentiated for 5‒7 days with the differentiation media. After decanting the differentiation media and washing with fresh culture media, the differentiated PC-12 cells on the coverslips were incubated for 1, 5, 10, and 15 min with a 1 μM recombinant peptides-containing solution in the culture media. After incubation, the cells were washed carefully three times with PBS, fixed with 3.7% w/v paraformaldehyde in PBS for 15 min, and permeabilized with 0.5% w/v Triton X-100 in PBS for 5 min. The nuclei and glycolipids/glycoproteins were stained with Hoechst 33258 (H3569; Invitrogen, Carlsbad, CA, USA) and WGA-AF647 (wheat germ agglutinin Alexa Fluor 647 conjugate; Invitrogen), respectively (15 min for each stain). The late endosomes were first labeled by treatment with an anti-RAB7 antibody (ab126712; Abcam, Cambridge, MA, USA) and second stained by treatment of an Alexa Fluor 594-conjugated antibody (A11012; Invitrogen) (1 h for each procedure). After washing three times with PBS, the coverslips were mounted with ProLong Gold Antifade Mountant (P10144; Molecular Probes, Eugene, OR, USA). The images were acquired using a Leica TCS SP8 HyVolution confocal microscope with a × 63 objective (HC PL APO 40×/1.10 W CORR CS2, FWD = 0.65 mm; Leica Microsystems, Wetzlar, Germany). All immunofluorescence images were acquired at 25 °C. The images presented together were processed identically. For the CLFM of MDCK cells, all procedures were identical to those of the PC-12 cells except MEM and non-coating coverslips.

The image processing was conducted with the Image J to quantify the green fluorescence of EGFP of the recombinant peptides adsorbed on the cells or internalized in the cells. The images merged were loaded on Image J, and only the green channel fluorescence in the images was collected using the macro (Supplementary Information). Subsequently, the outer line of the individual cell was selected manually based on the merged images. The mean values of the green fluorescence were then measured using the other macro (Supplementary Information) and fitted using regression curves [$mean=a\left(1-{b}^{t}\right)$; $a$ and $b$: constants; $t$: time (s)]. The increasing rate (${k}_{\mathrm{m}}$) of the mean values was determined at the initial point of the incubation as $-a\mathrm{ln}b$.

### Flow cytometry

In flow cytometry, while all the procedures before the incubation step with the recombinant peptides were the same as those for CLFM, the culture medium-based recombinant peptides (5 μM) were incubated for 2, 4, 8, and 16 min. After incubation, the cells were washed carefully three times with PBS and harvested by intensive pipetting > 100 times. The harvested cells were centrifuged (200 RCF, 3 min) and washed with PBS. The procedure was repeated three times. Subsequently, the cells re-suspended in PBS were loaded into a flow cytometer (guava easyCyte System; Millipore, MA, USA) to acquire the Green-B fluorescence signals of EGFP of the recombinant peptides within the cells. For data analysis, the obtained data files were loaded on FlowJo software (Version 10; FlowJo LLC, OR, USA). Based on the forward scatter-versus-side scatter plots, the signals only for cells (> 4000) were collected, and the signal histograms and mean values were acquired using the software. To quantify the recombinant peptides adsorbed on or internalized in the cells, the mean values acquired at 2, 4, 8, and 16 min were subtracted from a mean value of the cells and fitted using the regression curves [$\Delta mean=a\left(1-{b}^{t}\right)$]. The increasing rate (${k}_{\Delta \mathrm{m}}$) of the $\Delta mean$ was determined at the initial point of the incubation as $-a\mathrm{ln}b$.

### Statistical analyses

All data represents an average of at least three independent experiments or measurements. The results are reported as average ± standard deviation. The kinetic parameters and curves were determined and fitted using the regression iteration procedures in SigmaPlot 10.0 (IBM Co., Armonk, NY, USA). Statistical analyses were firstly started by examination of the data using the distribution normality test (Shapiro–Wilk test) and the variance homogeneity test (Levene’s test). If normal distribution and homogeneous variance were guaranteed, further statistical analyses (Tukey’s test) were conducted using SPSS Statistics (V23.0; IBM Co., Armonk, NY, USA).

## Supplementary Information

Supplementary Information.
