# Supplementary material for: Neuron-recognizable characteristics of peptides recombined using a neuronal binding domain of botulinum neurotoxin
Source: Sci Rep. 2022 Mar 23;12:4980. doi: 10.1038/s41598-022-09145-5 (PMC8943039; doi:10.1038/s41598-022-09145-5)
Supplement: Supplementary file 1 — Supplementary Information. [file 41598_2022_9145_MOESM1_ESM.pdf]

Neuron-recognizable characteristics of peptides recombined using a neuronal binding domain of botulinum neurotoxin

Hye Rin Kim[1†], Younghun Jung,[1,2†] Jonghyeok Shin,[5] Myungseo Park,[6] Dae-Hyuk Kweon[1,2,3,4]*, Choongjin Ban[7]*

[1]Department of Integrative Biotechnology, [2]Institute of Biomolecule Control, [3]Biologics Research Center, and [4]Interdisciplinary Program in BioCosmetics, Sungkyunkwan University, Seoburo 2066, Suwon, Gyeonggi 16419, Republic of Korea

[5]Carl R. Woese Institute for Genomic Biology, University of Illinois at Urbana-Champaign, Urbana, IL, 61801, USA

[6]Environmental Health Sciences, School of Public Health, University of Minnesota, Saint Paul, MN, 55108, USA

[7]Department of Environmental Horticulture, University of Seoul, 163 Seoulsiripdaero, Dongdaemun-gu, Seoul, 02504, Republic of Korea

*Corresponding authors:

Dae-Hyuk Kweon (Email: dhkweon@skku.edu) and Choongjin Ban (Email: pahncj@uos.ac.kr).

[†]These authors contributed equally to this study.

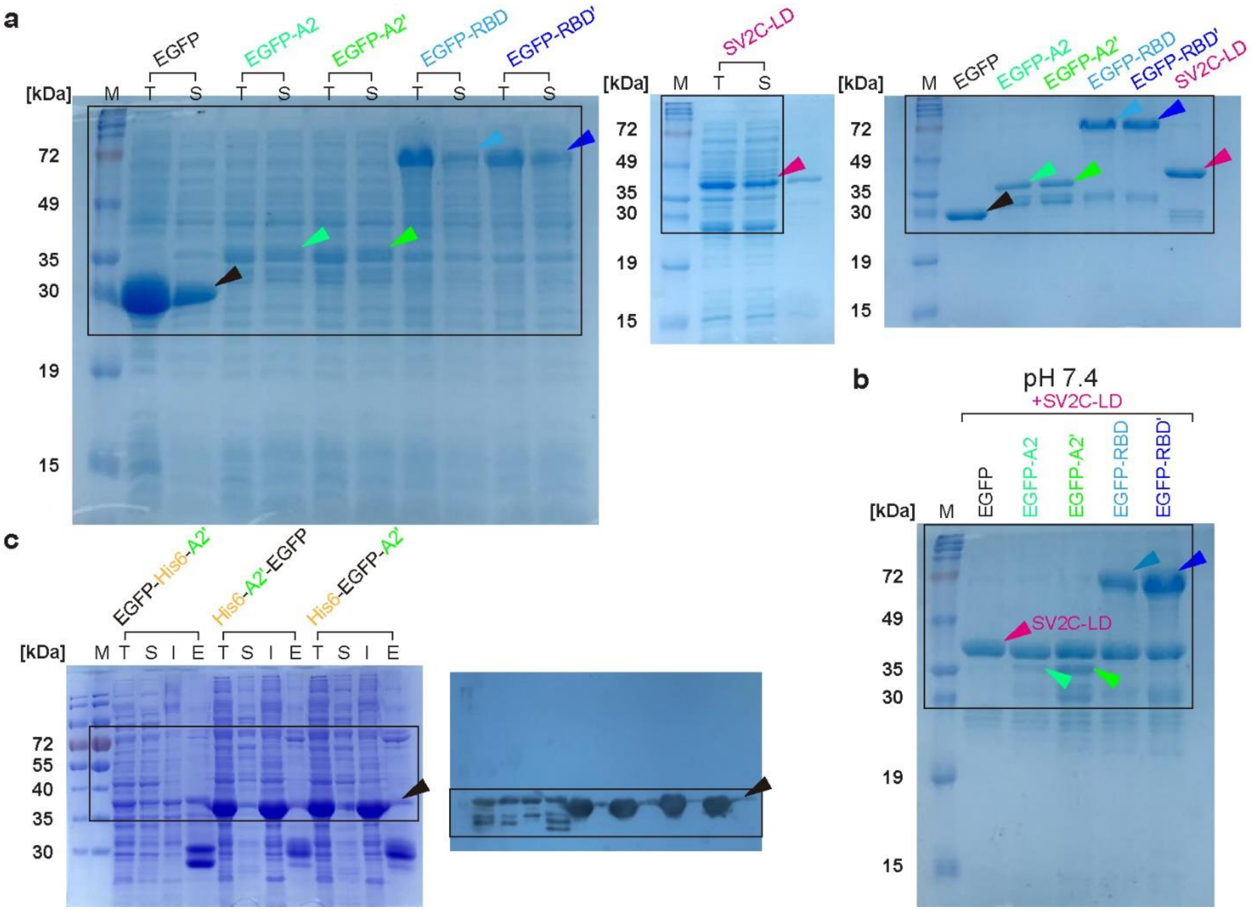

Fig. S1. Uncropped images for gel/blot analyses in Fig. 1, 2, and S7. (a) Uncropped gel images for Fig. 1c and 1d. (b) Uncropped gel images for Fig. 1c and 1d. (c) Uncropped gel images for Fig. S8b and S8c.

S1. Macros used for selecting the green channel and measuring the mean value

Selecting the green channel:

```
{if (nImages()!=1 || bitDepth()!=24)exit("Exactly one RGB image required"); id = getImageID(); run("Duplicate...", "title=Copy"); run("RGB Split"); selectWindow("Copy(red)"); run("Close"); selectWindow("Copy(blue)"); run("Close");selectImage(id);}
```

Measuring the mean value:

```
{if (nImages!=2)exit("Exactly 2 images required"); if (selectionType()==-1)exit("Selection required"); run("Put Behind [tab]"); run("Restore Selection"); run("Measure"); run("Put Behind [tab]");}
```

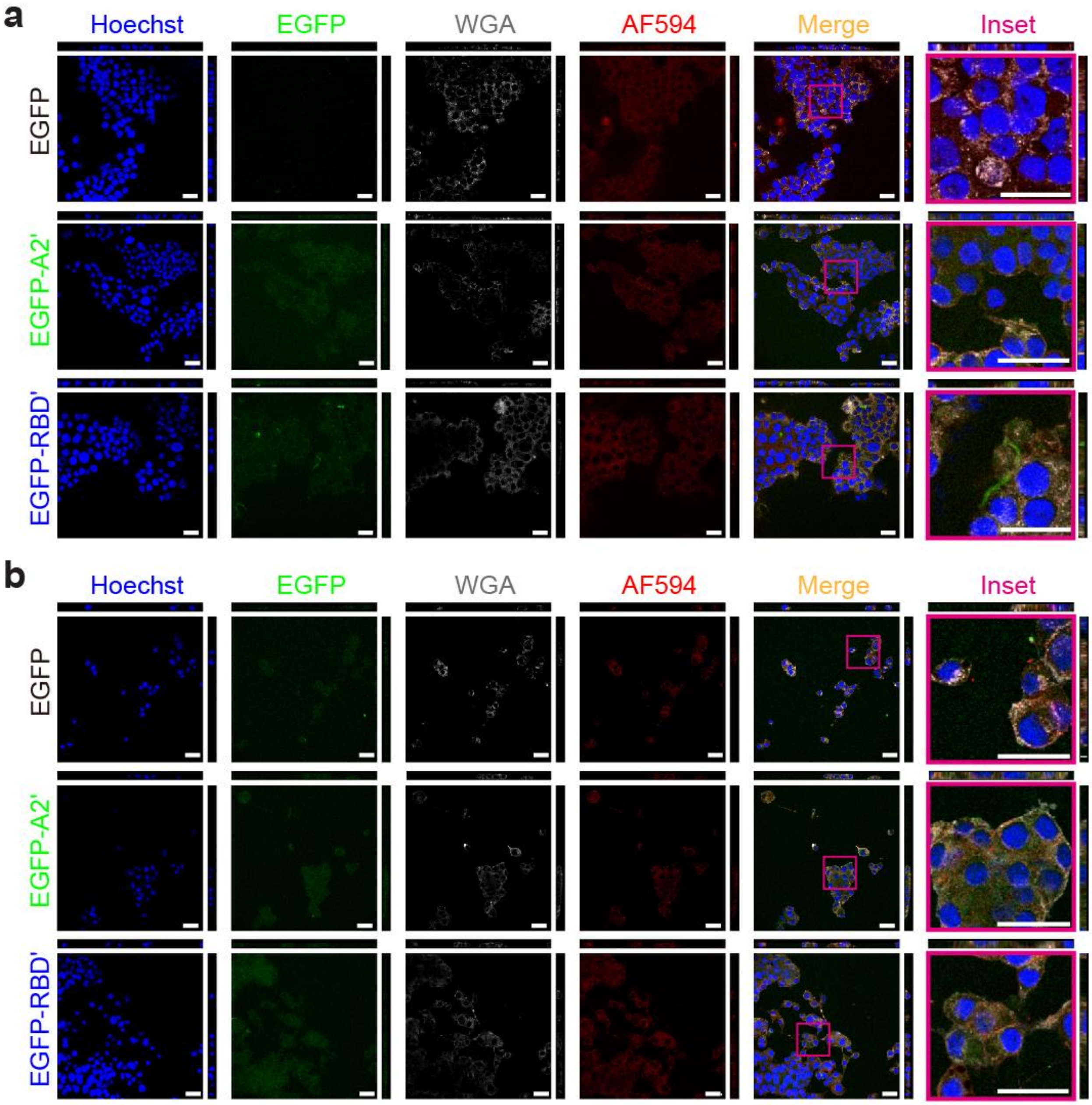

Fig. S2. Orthogonal-viewed images obtained by confocal laser fluorescence microscopy. Differentiated PC-12 cells incubated with culture media containing the recombinant peptides (EGFP, EGFP-A2′, and EGFP-RBD′) for (a) 1 or (b) 5 min (scale bars, 25 μm). Nuclei, EGFP, glycolipids/glycoproteins, and late endosomal proteins in the cells are in blue (Hoechst, Hoechst

33258), green (EGFP), gray (WGA, wheat germ agglutinin Alexa Fluor^{TM} 647 conjugate), and red (AF594, Alexa Fluor 594), respectively. The images at the bottom-left, top-left, and bottom-right in each image are the upper, y-axis, and x-axis views, respectively.

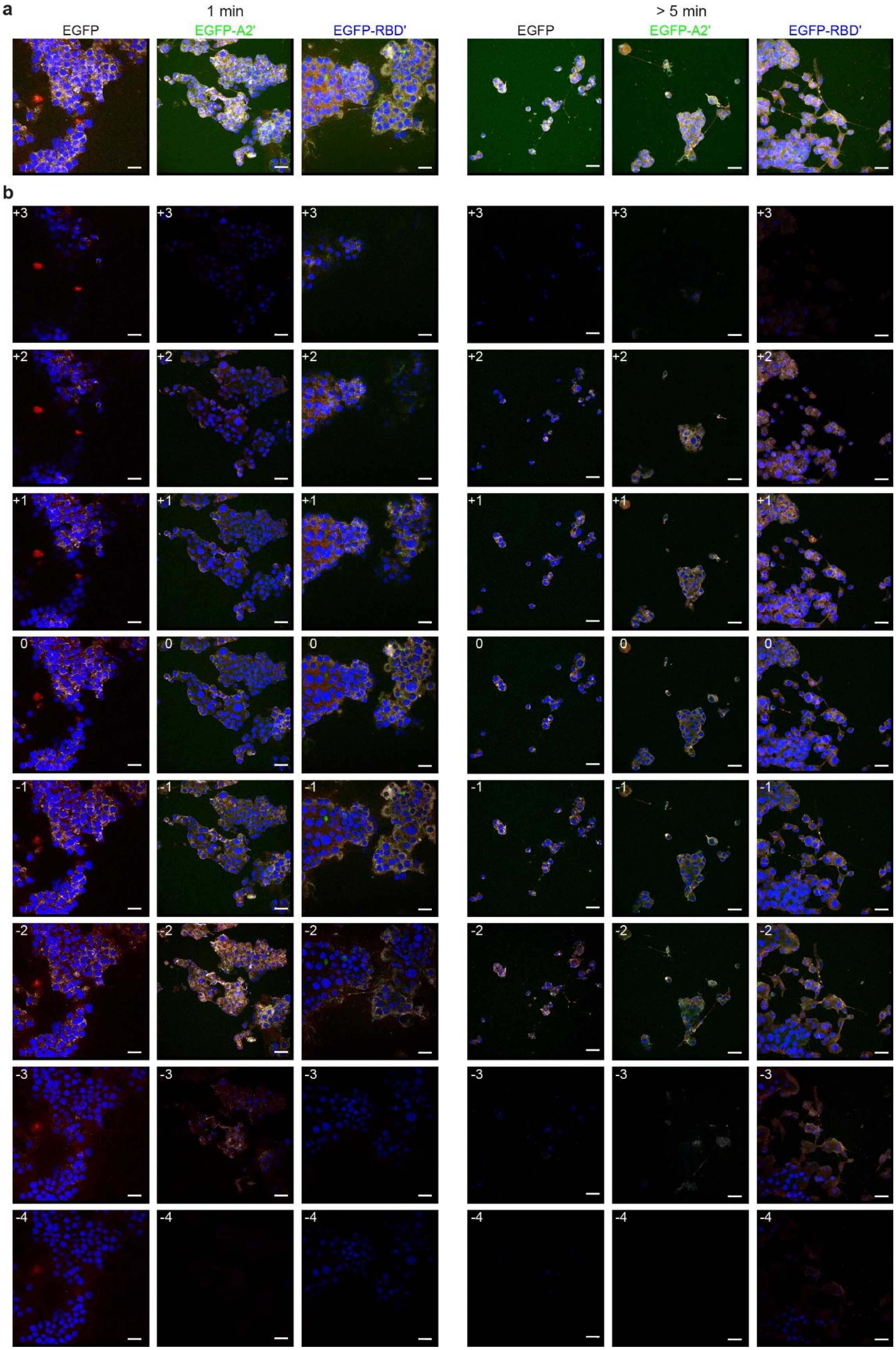
a
1 min
> 5 min
EGFP
EGFP-A2'
EGFP-RBD'
EGFP
EGFP-A2'
EGFP-RBD'
b
+3
+2
+1
0
-1
-2
-3
-4

Fig. S3. (a) Upper-view and (b) z-stack images obtained using confocal laser fluorescence microscopy. Differentiated PC-12 cells incubated with culture media containing the recombinant peptides (EGFP, EGFP-A2′, and EGFP-RBD′) for (a) 1 or (b) 5 min (scale bars, 25 μm). Nuclei, EGFP, glycolipids/glycoproteins, and late endosomes in the cells are in blue (Hoechst, Hoechst 33258), green (EGFP), gray (WGA, wheat germ agglutinin Alexa Fluor™ 647 conjugate), and red (AF594, Alexa Fluor 594), respectively. The z-stack images numbered for each section obtained along the z-axis are shown with an image taken at the mid-height of the nucleus defined as zero.

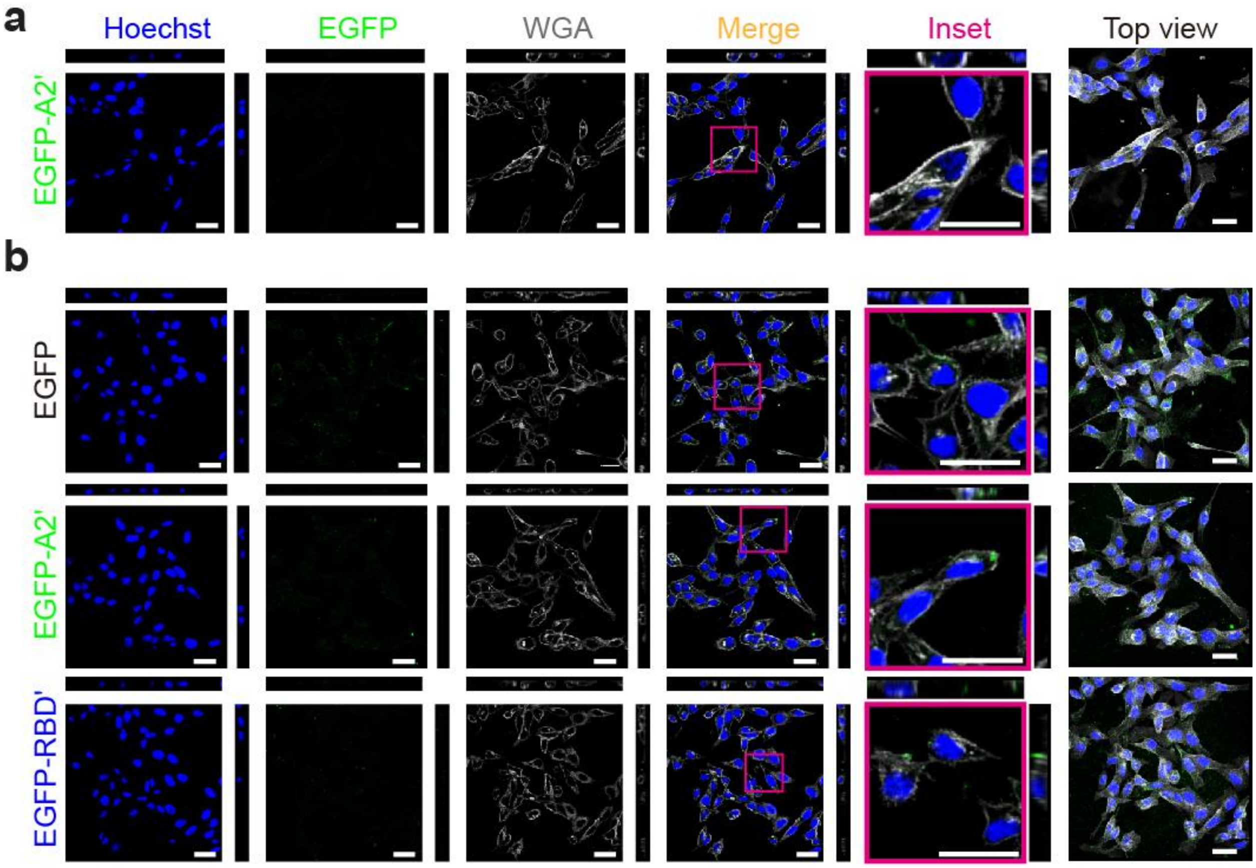

Fig. S4. Orthogonal-viewed and top view images obtained using confocal laser fluorescence microscopy. MDCK cells were incubated with culture media containing the recombinant peptides (EGFP, EGFP-A2′, and EGFP-RBD′) for (a) 1 or (b) 5 min (scale bars, 25 μm). Nuclei, EGFP, and glycolipids/glycoproteins in the cells are in blue (Hoechst, Hoechst 33258), green (EGFP), and gray (WGA, wheat germ agglutinin Alexa Fluor™ 647 conjugate), respectively. Images at the bottom-left, top-left, and bottom-right in each of the orthogonal-viewed images are the upper, y-axis, and x-axis views, respectively.

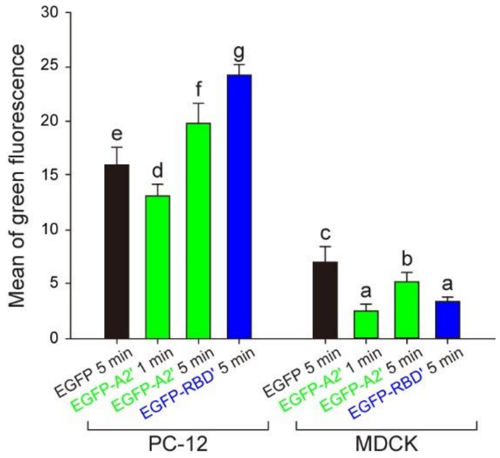

Fig. S5. Characterization of adsorption/absorption of the recombinant peptides to differentiated PC-12 cells and MDCK cells. Mean of green fluorescence in the cells incubated with culture media containing the recombinant peptides (EGFP, EGFP-A2′, and EGFP-RBD′), determined using the ImageJ processing of confocal laser fluorescence microscopic images. Data with different letters a–g in the plot represent significant differences according to the Tukey’s test ($n = 3$; average ± s.d.; $P < 0.05$).

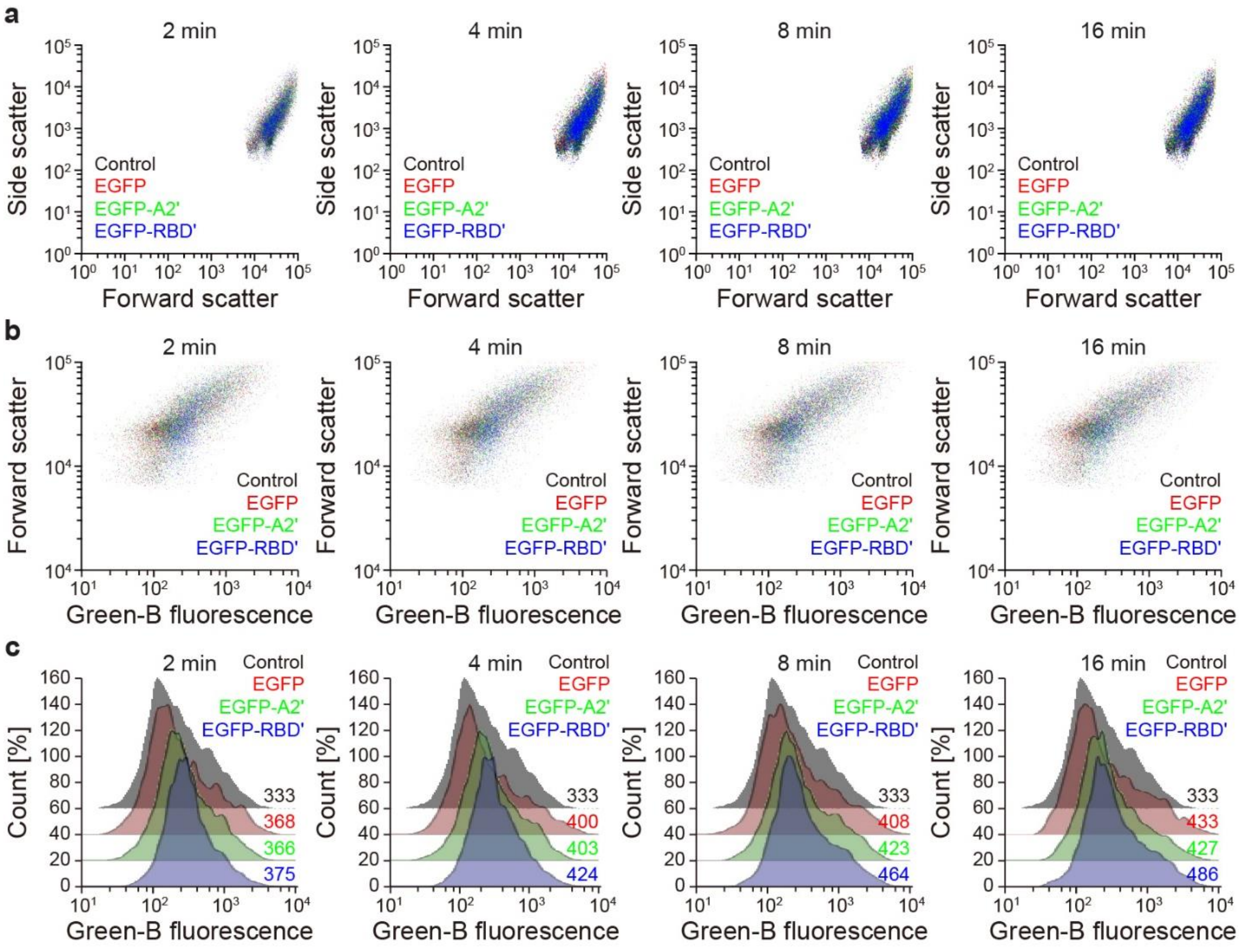

Fig. S6. Flow cytometry result. Differentiated PC-12 cells incubated with the culture media containing the recombinant peptides (EGFP, EGFP-A2′, and EGFP-RBD′). Scatter plots for (a) side scatter versus forward scatter, (b) forward scatter versus Green-B fluorescence, and (c) histograms for the Green-B fluorescence signal.

S2. Preparation of the primers and plasmid constructs

Plasmids pET28b-eGFP-His$_6$-linker-A$_2$ and -A$_2'$ were constructed by site-directed mutagenesis on pET28b-eGFP-His$_6$ using the forward (FW) and backward (BW) primers of eGFP-His$_6$-linker-A$_2$ and A$_2'$, respectively (Fig. S7a). The plasmid pET28b-His$_6$-eGFP-linker-A$_2'$ was constructed in two cloning steps (Fig. S7a). First, His$_6$ on plasmid pET28b-eGFP-His$_6$-linker-A$_2'$ was deleted by site-directed mutagenesis using the FW and BW primers of eGFP-His$_6$-linker-A$_2'$ del His$_6$. Second, His$_6$ was added on 5′ of the eGFP of the pET28b-eGFP-linker-A$_2'$ intermediate by site-directed mutagenesis using the FW and BW primers of His$_6$-eGFP-linker-A$_2'$. Plasmid pET28b-His$_6$-A$_2'$-linker-eGFP was constructed in three cloning steps (Fig. S7b). First, A$_2'$-linker was added to the 5′ of the eGFP of pET28b-eGFP-His$_6$ by site-directed mutagenesis using FW and BW primers of A$_2'$-linker-eGFP-His$_6$. Second, His$_6$ on plasmid pET28b-A$_2'$-linker-eGFP-His$_6$ was deleted by site-directed mutagenesis using FW and BW primers of A$_2'$-linker-eGFP-His$_6$ del His$_6$. Third, the His$_6$ was added to the 5′ of the A$_2'$ of pET28b-A$_2'$-linker-eGFP intermediate by site-directed mutagenesis using FW and BW primers of His$_6$-A$_2'$-linker-eGFP. Plasmid pET28b-eGFP-His$_6$-linker-Rbd was constructed in two cloning steps (Fig. S7c). First, a DNA fragment coding for the linker (four glycine + one serine) + BoNT/A1 RBD (Swiss-Prot Q7B8V4) was amplified with the FW and BW primers of linker-Rbd. Second, this DNA fragment was combined with a plasmid pET28b-eGFP-His$_6$, linearized with the FW and BW primers of pET28b-eGFP-His$_6$ V, using T4 DNA polymerase. The plasmid pET28b-eGFP-His$_6$-linker-Rbd′ was constructed from pET28b-eGFP-His$_6$-linker-Rbd in three sequential site-directed mutagenesis steps, using the FW and BW primers of eGFP-His$_6$-linker-RBD$^{1\prime}$, -RBD$^{2\prime}$, and -RBD$^{3\prime}$ at the first, second, and third steps (Fig. S7c). The lasmid pGEX-4T-1-Sv2cLd was constructed in two cloning steps (Fig. S7c). First, a DNA fragment coding for SV2C-LD was amplified with the FW and BW primers of Sv2cLd.

Second, this DNA fragment was ligated with a plasmid pGEX-4T-1, linearized using the FW and BW primers of pET28b-eGFP-His_6 V using T4 DNA polymerase. The sequences of the cloned plasmids were confirmed from Bionics Co., Ltd. (Seoul, Republic of Korea).

Table S1. Primers prepared and utilized in this study.

Primers	Sequences (5' → 3')
eGFP-His$_6$-linker-A$_2$ FW	GGTGGAGGCGGTAGCAGAGGGAATGTAATGACGACGAACATATACCTTAACAGCAGTTGAGATC CGGCTGCTAACAAAGCCCGAAA
eGFP-His$_6$-linker-A$_2$ BW	GTGGTGGTGGTGGTGGTGCTCG
eGFP-His$_6$-linker-A$_2$' FW	TTTCGGGCTTTGTTAGCAGCCGGATCTCAACTGCTGTTAAGGTATATGTTCGTCGTCATTACAGAC CCTCTGCTACCGCCTCCACC
eGFP-His$_6$-linker-A$_2$' BW	GTGGTGGTGGTGGTGGTGCTCG
A$_2$'-linker-eGFP-His$_6$ FW	GCTACCGCCTCCACCACTGCTGTTAAGGTATATGTTCGTCGTCATTACAGACCCTCTCATAGCTAC CATGGAACCGCGTGGCA
A$_2$'-linker-eGFP-His$_6$ BW	GTGAGCAAGGGCGAGGAGCTGTTC
A$_2$'-linker-eGFP-His$_6$ del His$_6$ FW	GAGCTTGTACAGCTCGTCCATGCCGA
A$_2$'-linker-eGFP-His$_6$ del His$_6$ BW	TGAGATCCGGCTGCTAACAAAGCCCG
His$_6$-A$_2$'-linker-eGFP FW	GCTACCGCCTCCACCGTGGTGGTGGTGGTGGTGCATAGCTACCATGGAACCGCGTGGC
His$_6$-A$_2$'-linker-eGFP BW	AGAGGGAATGTAATGACGACGAACATATACCTTAACAG
eGFP-His$_6$-linker-A$_2$' del His$_6$ FW	GAGCTTGTACAGCTCGTCCATGCCGA
eGFP-His$_6$-linker-A$_2$' del His$_6$ BW	GGTGGAGGCGGTAGCAGAGGGAAT
His$_6$-eGFP-linker-A$_2$' FW	GTGGTGGTGGTGGTGGTGGCTACCGCCTCCACCACTGCTGTTAAGGTATATGTTCGTCGTCATTAC ATTCC
His$_6$-eGFP-linker-A$_2$' BW	TGAGATCCGGCTGCTAACAAAGCCCGAAA
pET28b-eGFP-His$_6$ V FW	GCTACCGCCTCCACCGTGGTG
pET28b-eGFP-His$_6$ V BW	TGAGATCCGGCTGCTAACAAAGCCCGAAA
linker-Rbd FW	AGCAGCCGGATCTCACAGTGGGCGTTCGCCCCATCCAT
linker-Rbd BW	GGTGGAGGCGGTAGCAAAAACATTATTAATACTTCCATACTTAACCTGCGCTACGAGTCTAA
eGFP-His$_6$-linker-Rbd$^{1'}$ FW	TATGAATTTCGTGCCACGGTAAAGACTGCTGTT
eGFP-His$_6$-linker-Rbd$^{1'}$ BW	AACAGCAGTCTTTACCGTGGCACGAAATTCATA
eGFP-His$_6$-linker-Rbd$^{2'}$ FW	TTCGTCGTCATTACAGACCCTCTTGGGCCCTT
eGFP-His$_6$-linker-Rbd$^{2'}$ BW	AAGGGCCCAAGAGGGTCTGTAATGACGACGAA
eGFP-His$_6$-linker-Rbd$^{3'}$ FW	CACATTGTTTACGTCCACGTATTTGTTTGGGTC
eGFP-His$_6$-linker-Rbd$^{3'}$ BW	GACCCAAACAAA ACGTGGACGTAAACAATGTG
pGEX-4T-1 V FW	CTGACGATCTGCCTCGCGCGTTTC
pGEX-4T-1 V BW	TCAGTCACGATGCGGCCGCTCGA
Sv2cLd FW	CCGCATCGTGACTGA GACGTGATAAAGCCCCTTCAGTCAGATGAATATG
Sv2cLd BW	GAGGCAGATCGTCAGATCGAAAGTAATCTGGCAGCCGGTCTTATTATGAAAG

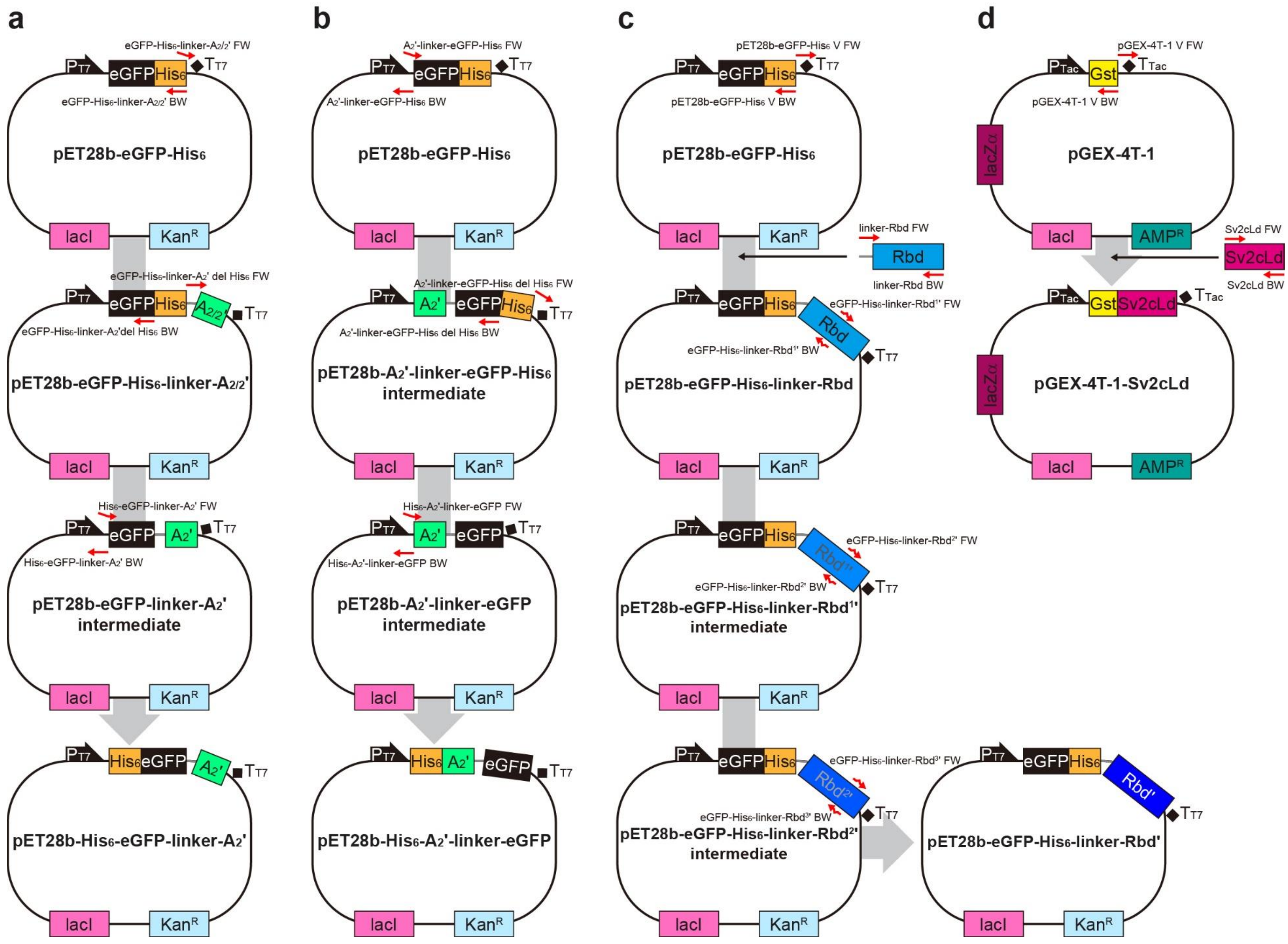

a
b
c
d
pET28b-eGFP-His6
pET28b-eGFP-His6-linker-A2/2'
pET28b-eGFP-linker-A2' intermediate
pET28b-His6-eGFP-linker-A2'
pET28b-A2'-linker-eGFP-His6 intermediate
pET28b-A2'-linker-eGFP intermediate
pET28b-His6-A2'-linker-eGFP
pET28b-eGFP-His6-linker-Rbd
pET28b-eGFP-His6-linker-Rbd1' intermediate
pET28b-eGFP-His6-linker-Rbd2' intermediate
pET28b-eGFP-His6-linker-Rbd'
pGEX-4T-1
pGEX-4T-1-Sv2cLd
eGFP-His6-linker-A2/2' FW
eGFP-His6-linker-A2/2' BW
eGFP-His6-linker-A2' del His6 FW
eGFP-His6-linker-A2'del His6 BW
His6-eGFP-linker-A2' FW
His6-eGFP-linker-A2' BW
A2'-linker-eGFP-His6 FW
A2'-linker-eGFP-His6 BW
A2'-linker-eGFP-His6 del His6 FW
A2'-linker-eGFP-His6 del His6 BW
His6-A2'-linker-eGFP FW
His6-A2'-linker-eGFP BW
pET28b-eGFP-His6 V FW
pET28b-eGFP-His6 V BW
linker-Rbd FW
linker-Rbd BW
eGFP-His6-linker-Rbd1' FW
eGFP-His6-linker-Rbd1' BW
eGFP-His6-linker-Rbd2' FW
eGFP-His6-linker-Rbd2' BW
eGFP-His6-linker-Rbd3' FW
eGFP-His6-linker-Rbd3' BW
pGEX-4T-1 V FW
pGEX-4T-1 V BW
Sv2cLd FW
Sv2cLd BW
PT7
TT7
PTac
TTac
eGFP
His6
A2/2'
A2'
Rbd
Rbd1'
Rbd2'
Rbd'
Gst
Sv2cLd
lacI
KanR
AMPR
lacZα

Fig. S7. Schematic diagram of the construction of (a) pET28b-eGFP-His_6-linker-A_2, pET28b-eGFP-His_6-linker-A_2', and pET28b- His_6-eGFP-linker-A_2', (b) His_6-A_2'-linker-eGFP, (c) pET28b-eGFP-His_6-linker-Rbd, pET28b-eGFP-His_6-linker-Rbd′, and (d) pGEX-4T-1-Sv2cLd.

S3. Immunoblotting

After SDS-PAGE, the peptides for the gel bands were transferred to a polyvinylidene difluoride membrane and incubated in 25 mM Tris-HCl buffer containing 0.9% w/v NaCl, 0.05% w/v Tween 20, and 5% w/v skim milk (1 h, 25°C, and pH 7.5). Subsequently, the membrane was incubated overnight at 4°C in a buffer containing the anti-polyhistidine-peroxidase antibody (A7058; Sigma Aldrich Co., St. Louis, MO, USA), diluted 1,000-fold, and washed three times with the buffer except for skim milk (TBST). After re-incubation in TBST containing horseradish peroxidase-conjugated rabbit anti-immunoglobulin G diluted 4,000-fold (1 h and 25°C), the membrane was washed three times with TBST and developed in an enhanced chemiluminescence solution.

S4. Improvement of proteolytic stability of the neuro-recognizable recombinant peptides

Even after the purification procedures, in a gel lane of EGFP-A2′ (Fig. 1d), relatively dark bands not corresponding to the target peptide were still observed at ~35 kDa, indicating proteolysis. The recombinant gene construct as eGFP-His$_6$-linker-A$_2$′ was redesigned by changes in the order of eGFP, His$_6$, linker, and A$_2$′, to optimize the construct in terms of the stability against the proteolysis; herein, the target peptide is referred to as EGFP-His6-A2′. The other two recombinant gene constructs (Fig. 3a) were prepared as His$_6$-A$_2$′-linker-eGFP and His$_6$-eGFP-linker-A$_2$′ to express His6-A2′-EGFP and His6-EGFP-A2′ (Fig. S1a and b), respectively. In the SDS-PAGE gel image (Fig. 3b), the bands for the lyzed residues were barely observed in both lanes of His6-A2′-EGFP and His6-EGFP-A2′, irrespective of their solubility and the elution, unlike those for EGFP-His6-A2′. In the anti-polyhistidine-immunoblotting image (Fig. 3c), no band for the lyzed peptides was observed in the lanes of His-A2′-EGFP and His-EGFP-A2′. This suggests improved proteolytic stability. Moreover, dark and thick target bands were detected in the total lanes of His6-A2′-EGFP and His6-EGFP-A2′. In terms of the relative amount values for the total lanes (Fig. 3d), the reconstructed target peptides (His6-A2′-EGFP and His6-EGFP-A2′) were expressed > 2.7-times more than the original peptide (EGFP-His6-A2′). This result suggests that the construct redesigning reduced the proteolysis and increased the yield of target recombinant peptides.

According to the three-dimensional structure model of EGFP (Swiss-Prot A0A348GST9), each of the *N*- and *C*-termini was located separately at opposite sites on an upper plane of the barrel (Fig. S2). In this regard, His6-EGFP-A2′ rather than His6-A2′-EGFP is a better construct to avoid the structural interruption of His6- and A2′ and conserve their respective functions, *i.e.*, tagging and neuro-recognizing functions. Therefore, His6-EGFP-A2′ was used for the experiments as the association/dissociation kinetics and cell internalization. The insoluble His6-EGFP-A2′ was

expressed > 5.5-times more abundantly than the soluble His6-EGFP-A′ (Fig. 3d). Therefore, the insoluble His6-EGFP-A′ was used for the experiments below after solubilization and refolding.

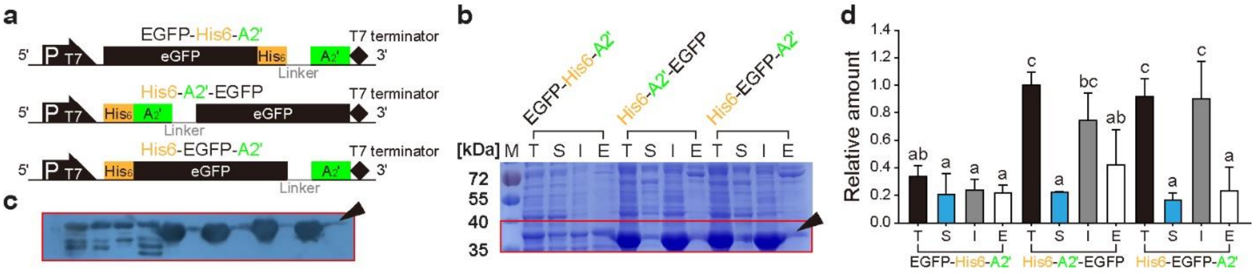

Fig. S8. Optimization of the recombinant constructs against proteolysis. (a) Schematic structural illustrations of the gene constructs (eGFP-His$_6$-linker-A$_2$′, His$_6$-A$_2$′-linker-eGFP, and His$_6$-eGFP-linker-A$_2$′) used to express the recombinant peptides (EGFP-His6-A2′, His6-A2′-EGFP, and His6-EGFP-A2′; linker sequence: GGGGS). (b) Gel and (c) anti-polyhistidine-immunoblotting images of the recombinant peptides for the SDS-PAGE, obtained after centrifugation and elution using Ni-NTA resin-filled column (M, marker peptide; T, Total peptide; S, soluble peptide; I, insoluble peptide; E, peptide eluted from a purifying column; an arrow indicates the target peptides). (d) Relative amounts of the target peptides measured using the ImageJ processing with the gel images, based on the concept that the total His6-A2′-EGFP is 1. Data with different letters a–c in the plot represent significant differences according to the Tukey's test ($n \geq 3$; average ± s.d.; $P < 0.05$). Original gels/blots are presented in Supplementary Fig. S9.

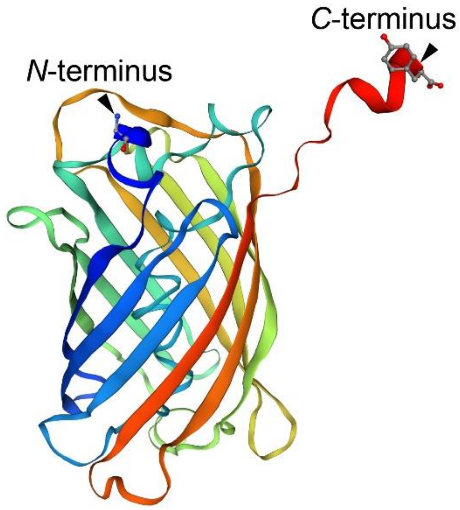

Fig. S9. 3-dimensional structure model of the EGFP (Swiss-Plot A0A348GST9) obtained from https://swissmodel.expasy.org/repository/uniprot/A0A348GST9?csm=B4457D791D29352F; the arrows indicate *N*- and *C*-termini of the EGFP.
